# Polymeric micelles with dual thermal and reactive oxygen species (ROS)-responsiveness for inflammatory cancer cell delivery

**DOI:** 10.1186/s12951-017-0275-4

**Published:** 2017-05-16

**Authors:** Meiqiong Tang, Ping Hu, Qiang Zheng, Nicola Tirelli, Xiaohong Yang, Zhanlong Wang, Yanfang Wang, Qing Tang, Yun He

**Affiliations:** 1 School of Pharmaceutical Sciences, Chongqing University, 55 South Daxuecheng Road, Chongqing, 401331 China; 2 NorthWest Centre of Advanced Drug Delivery (NoWCADD), School of Pharmacy, and Centre for Tissue Injury and Repair, Institute of Inflammation and Repair, University of Manchester, Oxford Road, Manchester, M13 9PT UK; 3 First Affiliated Hospital of the Medical College, Shihezi University, Xinjiang, 832008 People’s Republic of China

**Keywords:** Nanomedicine, Polymeric micelle, ROS-responsive, Cellular uptake

## Abstract

**Background:**

The object of this study was to develop a thermally and reactive oxygen species-responsive nanocarrier system for cancer therapy.

**Results:**

PPS-PNIPAm block copolymer was designed and synthesised using a combination of living anionic ring-opening polymerization and atom transfer radical polymerization. The synthesized polymer formed micellar aggregates in water and demonstrated dual responsiveness towards temperature and oxidants. Using doxorubicin (DOX) as a model drug, encapsulation and in vitro release of the drug molecules in PPS-PNIPAm nanocarriers confirmed the responsive release properties of such system. Cell uptake of the DOX loaded micelles was investigated with human breast cancer cell line (MCF-7). The results showed Dox-loaded micelles were able to be taken by the cells and mainly reside in the cytoplasma. In the stimulated cells with an elevated level of ROS, more released DOX was observed around the nuclei. In the cytotoxicity experiments, the Dox-loaded micelles demonstrated comparable efficacy to free DOX at higher concentrations, especially on ROS stimulated cells.

**Conclusions:**

These results demonstrated that PPS-PNIPAm nanocarriers possess the capability to respond two typical stimuli in inflammatory cells: temperature and oxidants and can be used in anticancer drug delivery.

**Electronic supplementary material:**

The online version of this article (doi:10.1186/s12951-017-0275-4) contains supplementary material, which is available to authorized users.

## Background

Cancer nanomedicine has emerged as a promising approach to deliver anticancer therapeutics to tumors through enhanced permeability and retention (EPR) effect [1–4]. Among the various developed nanoscale drug delivery systems, stimuli-responsive drug delivery systems attracted ever-increasing attention in recent years owing to their “smart” capabilities to precisely deliver cargos to the desired cancer sites compared to the conventional passive targeting approaches, resulting in improved treatment and minimised systemic toxicity [5–7]. In the midst of these stimuli responsive systems, oxidation-responsive mechanisms are extremely attractive for selectively triggering the release of hydrophobic drug molecules from the nanocarriers due to often observed oxidants overproduction associated with a number of specific diseases [8–12].

In human body, oxidants such as reactive oxygen species (ROS) or reactive nitrogen species (RNS) play key roles as either signalling molecules in a broad range of physiological processes including apoptosis, immunity, and so forth [13–16]. However, oxidative stress induced by ROS overproduction leads to a broad array of chemical biomolecule modification causing various diseases such as inflammatory disorders, diabetes and cancers [17–20]. To date, nevertheless, there are relatively limited number of oxidation-responsive systems, in comparison e.g. to those responding stimuli such as acidic pH, reductive potential, light etc. [5, 21–23].

**Scheme 1:**
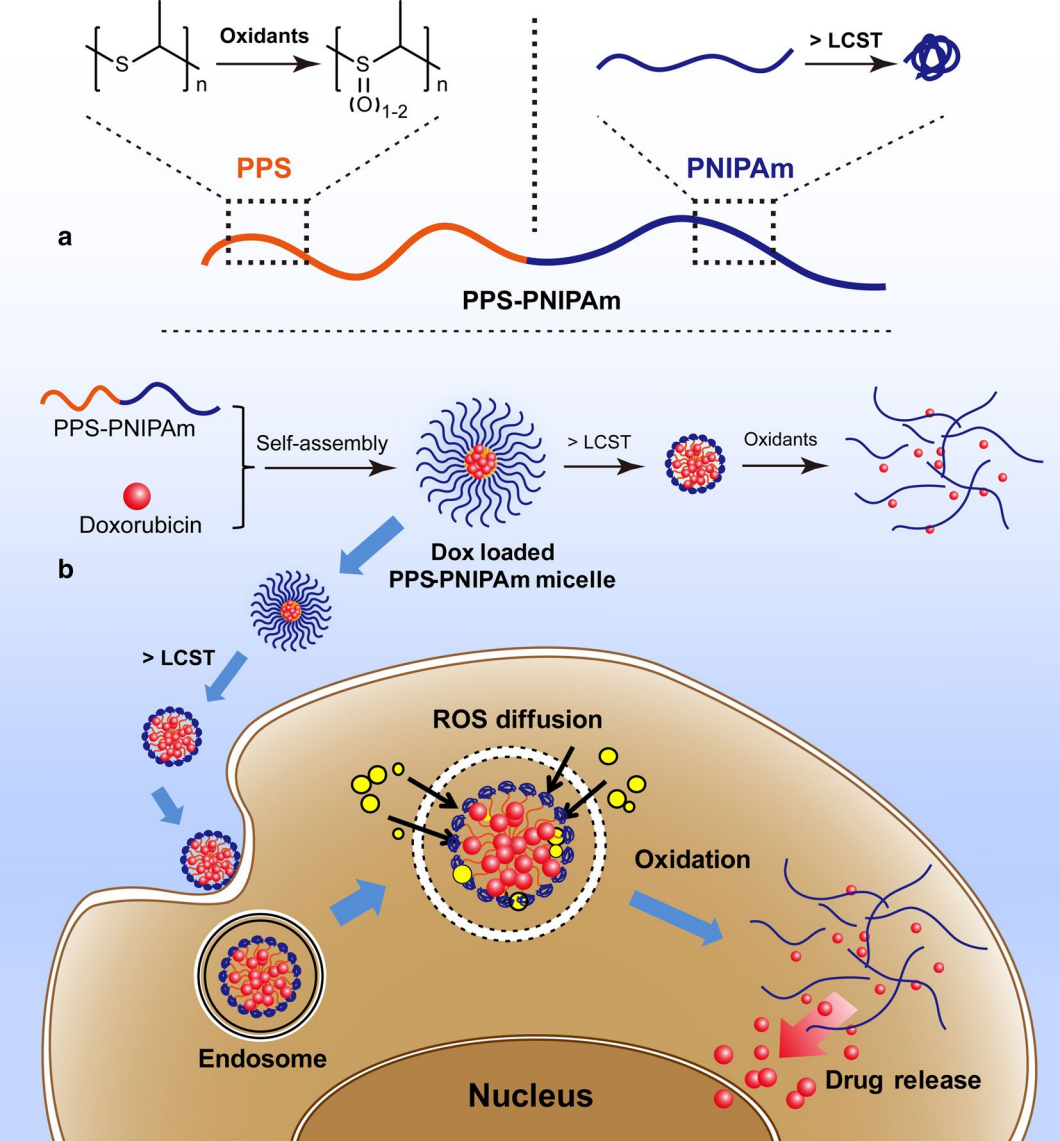
Illustration of the stimuli-responsiveness of PPS-PNIPAm block copolymer. **a** Oxidation-responsiveness of PPS block and temperature-responsiveness of PNIPAm block. **b** Self-assembling of PPS-PNIPAm micelles and loading with doxorubicin. The PNIPAm corona undergoes shrinkage and collapse above its LCST and PPS core can be furtherly oxidised and release encapsulated DOX molecules. Such drug-loaded nanocarrier can be used in cancer cell delivery and release drug in ROS intracellular environment

Poly(propylene sulfide) (PPS) is at the basis of some popular ROS-sensitive constructs [24]; this polymer, features a thioether in each repeating unit, which enables its response to be based on a hydrophobic (thioether)-to-hydrophilic (sulfoxide, sulfone) transition (Scheme 1a) [8, 25–28]. Correspondingly, nanocarriers featuring PPS as the hydrophobic domain have been explored as ROS- and inflammation-responsive systems [9, 25, 29].

In our study, such a concept has been advanced by combining PNIPAm [30, 31], a well-known thermally responsive polymer to the PPS block. This approach aims to create a diblock copolymer PPS-PNIPAm, that is responsive to two different stimuli: oxidants and higher temperature, which are typically found in inflammation and also in tumoural tissues [32, 33]. It is assumed the nanocarriers, with PPS cores and PNIPAm corona in water, could undergo a corona collapse at higher temperature owing to the disruption of hydrogen bonding of the PNIPAm with the water molecules [34]. Such temperature-induced morphological change has been reported by using direct heating, microwave radiation or near infrared light as the source of stimuli [35–38]. Combining PNIPAm to PPS block would bring advantages to the previously reported PPS-PEG nanocarrier systems: (a) above the LCST of PNIPAm, the shrinkage of PNIPAm corona would favour the retention of such drug carriers in tumour sites through EPR effect due to reduced sizes of nanocarriers. (b) Furthermore, the shrunk hydrophilic corona would allow easier access of ROS to reach the hydrophobic PPS core, therefore enhance the oxidation-responsiveness of the system (Scheme 1b).

In this study, PPS-PNIPAm block copolymers were synthesised by combining a living anionic ring opening polymerisation for PPS and an atom transfer radical polymerisation (ATRP) for PNIPAm [39, 40]. The preparation of polymeric micelles, the drug release upon applied stimuli, the uptake and efficacy of drug loaded micelles with MCF-7 cell lines were systemically investigated. Especially, we focused on the influence of ROS to the cellular uptake, intracellular drug release and cytotoxicity of anticancer drug loaded PPS-PNIPAm micelles. The overall investigation revealed that the newly developed dually responsive polymeric drug delivery system is very promising for temperature and ROS overproduction related cancer treatment.

## Methods

### Materials

2-Mercaptotethanol (>99%), tris-*n*-butylphosphine (TBP), propylene sulfide (PS, 98%), 1,8-diazabicyclo [5.4.0] undec-7-ene (DBU, 98%), acetic anhydride (98.5%), ethyl bromide acetate (EBA, 98%), 2-bromo-2-methylpropionylbromide (BrBiB, 98%), *N*-isopropyl acrylamide (NIPAm, 98%), copper (I) bromide (CuBr, >99.99%), N,N,N′,N″,N″-pentamethyl diethylenetriamine (PMDETA, >98%), doxorubicin hydrochloride (DOX·HCl), hydrogen peroxide (H_2_O_2_) were purchased from Sigma (St. Louis, USA). Cell Counting Kit-8 (CCK-8), Reactive Oxygen Species (ROS) Assay Kit, Hoechst 33258 were purchased from Beyotime Institute of Biotechnology (Beijing, China).

### Physico-chemical characterisation

^1^H NMR spectra were obtained using a Bruker Avance 400 MHz instrument (Switzerland). CDCl_3_ was used as the solvent. The mean particle size and ζ-potential of micellar aggregates were determined by dynamic light scattering (DLS) using a Malvern Zetasizer Nano ZS90 (Malvern, UK). DLS samples (micelles with/without DOX-loaded) were prepared in water, and was filtered using a 0.22 μm Nylon Syringe Filter prior to the measurements. The molecular weight distribution was determined by gel permeation chromatography (GPC) equipped with a 1260 Infinity Isocratic Pump and an RI detector (Agilent, US). DMF containing 0.1 mol% LiBr was the elute and the flow rate was 1.0 mL/min. Linear poly(methyl methacrylate) standards from Fluka were used for calibration. FT-IR spectra were recorded in ATR mode (Golden gate) on a Tensor 27 Bruker spectrometer (Germany). The particles were imaged using a Tecnai G2 F20 TWIN transmission electron microscope operated at 200 kV and equipped with a field-emission gun (FEI, Netherland). The sample was placed onto a Quantifoil grid, followed by utilizing Vitrobot, and then flash frozen in liquid ethane. The images were recorded at magnification of 14,500 and 25,000 with a 4 K * 4 K eagle CCD camera and defocus ranging from 2 to 3 μm. Confocal images of the samples were taken using the Leica TCS SP5 (Germany).

### Polymer synthesis

*PPS-Br ATRP macroinitiator* PPS-OH polymer was synthesized according to a previously reported method [9, 41]. Degassed THF (5 mL) was transferred to the reactor and then TBP (5.0 equiv) was added dropwise. PPS-OH (0.29 mmol) was dissolved in 15 mL of anhydrous THF followed by addition of anhydrous triethylamine (55 μL, 0.39 mmol) under nitrogen atmospheres. The reaction flask was then transferred into an ice-salt bath and 2-bromoisobutyryl bromide (0.17 mL, 1.3 mmol) was added drop wise. The reaction mixture was stirred for 24 h at room temperature. Finally, the solution was concentrated and precipitated in cold methanol. The precipitated PPS-Br was centrifuged and dried under vacuum to constant weight with yield of 82%. ^1^H-NMR (CDCl_3_): 1.2–1.35 (t, 3H, C**H**_**3**_CH_2_–), 1.35–1.45 (d, –C**H**_**3**_ in PPS chain), 1.9 (s, 6H, –C**H**_**3**_), 2.55–2.75 (m, 1 diastereotopic H of –C**H**_**2**_– in PPS chain), 2.6 (t, 2H, OH–CH_2_–C**H**_**2**_–S–), 2.85–3.05 (m, –C**H–** and 1 diastereotopic H of –C**H**_**2**_– in PPS chain), 3.3 (t, 2H, OH–C**H**_**2**_–CH_2_–S–), 3.7 (s, 1H,O**H**–), 4.25–4.35 (m, 2H, CH_3_C**H**_**2**_–). FT-IR (KBr): 2960 (*ν*_as_ CH_3_), 2922 (*ν*_as_ CH_2_), 2868 (*ν*_s_ CH_3_ and *ν*_s_ CH_2_), 1736 (*ν* C=O ester), 1451 (CH_2_), 1373, 1275, 1225, 1175, 1102 (*ν* C–O–C) cm^−1^.

*PPS-PNIPAm* Bifunctional 2-bromopropionate PPS macroinitiator (0.64 mg, 0.33 mmol), NIPAm (1.49 g, 13.2 mmol), THF (250 mL) and CuBr (104.1 mg, 0.73 mmol) were dissolved in a two-neck flask and the mixture was degassed for 1 h. Then 580 μL of PMDETA was injected and the mixture was allowed to react for another 8 h at room temperature. The reaction was terminated by exposing the reaction mixture to air. The solution was passed through a short aluminum oxide column to remove the catalyst complex. The polymer was obtained with 48% yield after precipitation in cold diethyl ether, dialysis and vacuum drying. ^1^H-NMR (CDCl_3_): 1.19 (m, –NHCH(C**H**_**3**_)_2_ in PNIPAm), 1.35–1.45 (d, –C**H**_**3**_ in PPS chain), 1.58–2.38 (m, –C**H**– and –C**H**_**2**_– in PNIPAm), 2.55–2.75 (m, 1 diastereotopicH of –C**H**_**2**_– in PPS chain), 2.85–3.05 (m, –C**H**– and 1 diastereotopic H of –C**H**_**2**_– in PPS chain), 5.8–7.0 (m, –N**H**CH(CH_3_)_2_ in PNIPAm), 4.01 (m, –C**H**(CH_3_)_2_ in PNIPAm). FT-IR (KBr):1645 (amide I, *ν* C=O), 1546 (amide II, δ N–H), 1387 (*ν*_s_, –CH_3_), 1171 (*ν* C–C) cm^−1^.

### Preparation of PPS-PNIPAm micelles

PPS_10_-PNIPAm_40_ (4 mg) were dissolved in 1 mL of acetone. Using an automatic syringe, the organic solution was slowly (0.1 mL/min) injected to 10 mL of deionised water with a magnetic stirring bar at room temperature. After stirring for 20 min, the organic solvent was removed by a rotary evaporator (80 mbar for 40 min, T = 25 °C). Finally, the dispersion was put in a dialysis tube (MWCO = 8000–14,000) and dialyzed against distilled water for 24 h.

### Drug loading

Doxorubicin-loaded micelles were prepared as follows: 10 mg of DOX·HCl was added into 20 mL of freshly prepared PPS-PNIPAm micelles (1 mg/mL), then 1.5 molar equiv of triethylamine was added to the micellar dispersion and stirred at room temperature for 12 h to reach equilibrium. The unloaded free drug and the salt produced by neutralization reaction were removed using a dialysis tube (Mn 8000–14,000) against 3000 mL of pure water with stirring at room temperature. Pure water was replaced for every 5 h. Drug loading (DL) and encapsulation efficiency (EE) were calculated as follows: DL (w/w) = (amount of loaded drug)/(amount of polymer), EE (wt%) = (actual amount of loaded drug)/(theoretical amount of loaded drug).

### In vitro drug release

The temperature and H_2_O_2_ responsive release of the DOX experiments were carried out using DOX-loaded micelles (1 mg/mL). Two millilitre of sample was placed in a glass bottle and 8.0 mL of pH 7.4 PBS buffer was added. Two aliquots of the resultant mixture was stirred slowly at 25 and 37 °C, respectively. The H_2_O_2_-triggered cargo release was achieved by adding the H_2_O_2_ (0.1%) to the DOX-loaded solution. An excitation beam of 480 nm was directed into the solution to excite the fluorescent emission of the released DOX. The emission of fluorescence was measured at 540 nm at 1, 2, 4, 6, 8, 10 h. The released percentage of DOX was calculated as (F_t_ − F_0_)/(F_f_ − F_0_) × 100%, where F_t_ represents the fluorescence reading of sample at time t, F_0_ represents the fluorescence reading of sample at time 0, F_f_ represents the fluorescence reading of the free DOX in PBS with the same DOX concentration as the DOX loaded sample. Release profiles were obtained by plotting the release percentage versus time.

### ROS stimulation of different cells

Before verifying the influence of ROS on the uptake and release of the DOX with cells, intracellular ROS level was measured using a commercialized ROS assay kit in accordance with the manufacturer’s instructions (Beyotime Institute of Biotechnology, China). Briefly, after seeding the cells (L-02 and MCF-7 cell lines) in a 24-well plate at 3 × 10^4^ cells/well and incubated for 24 h in 0.5 mL of DMEM medium with 10% PBS. Cells were pre-treated with Rosup reagent (50 μg/mL) for 20 min and then incubated with DCFH-DA (10 mM) for 30 min and washed off. The cells were observed by an inversed fluorescent microscope and imaged to check the fluorescence intensity which represented the amounts of intracellular ROS level.

### In vitro cellular uptake

The in vitro cellular uptake was performed on MCF-7 cell line. The cells were seeded on glass coverslips in a 24-well plate at 3 × 10^4^ cells/well and incubated for 24 h in 0.5 mL of DMEM medium with 10% PBS. The DOX-loaded micelles were added to each well and incubated with the cells for 1, 2 and 4 h at 37 °C. To compare the effect of ROS to the uptake and intracellular DOX release, cells pre-treated with Rosup reagent (50 μg/mL) for 20 min were used as ROS-stimulated cells to evaluate such effect. After incubation, the supernatant was carefully removed and then the cells were fixed with 4% formaldehyde for 20 min at room temperature. After the washing steps, the nuclei were stained with Hoechst 33258 in PBS. The cells were observed with an inversed fluorescent microscope and a confocal laser scattering microscope (CLSM). The fluorescence intensity of each cell was processed and calculated by Photoshop software (Adobe, CA, USA).

### Biocompatibility and efficacy studies

The biocompatibility of PPS-PNIPAm polymer was evaluated using the CCK-8 assay on MCF-7 and A549 cell lines. The cells were seeded in a 96-well plate (5 × 10^4^ cells/well) for overnight, treated with various concentrations of polymers, were added to a DMEM medium containing 10% fetal bovine serum (FBS) in a humidified 37 °C incubator supplied with 5% carbon dioxide and incubated for 48 h. After removal of the culture media from cell culture plates, 100 μL of fresh culture media and 10 μL of CCK-8 kit solutions were immediately added and homogeneously mixed followed by 2 h incubation. The optical density of each well at 450 nm was read by a microplate reader. Cells cultured in DMEM medium containing 10% FBS (without exposure to polymers) were used as controls.

The cytotoxicity of DOX loaded PPS-PNIPAm micelles was evaluated using the same procedure. After seeded in the plate for overnight, MCF-7 cells were treated with various concentrations of DOX-loaded micelle samples instead of polymer dispersions.

### Statistical analysis

All results are expressed as mean ± SEM. Prism software (GraphPad) was used for statistical analysis. Statistical comparisons were performed by unpaired Student’s *t* test when 2 groups of equal variance were compared and by One-way ANOVA with post hoc tests with the Bonferroni correction when >2 groups were compared. Probability values <0.05 were considered statistically significant (NS: not significantly different, *p < 0.05, **p < 0.01, ***p < 0.001).

## Results and discussion

### Synthesis and characterizations of PPS-PNIPAm

**Fig. 1:**
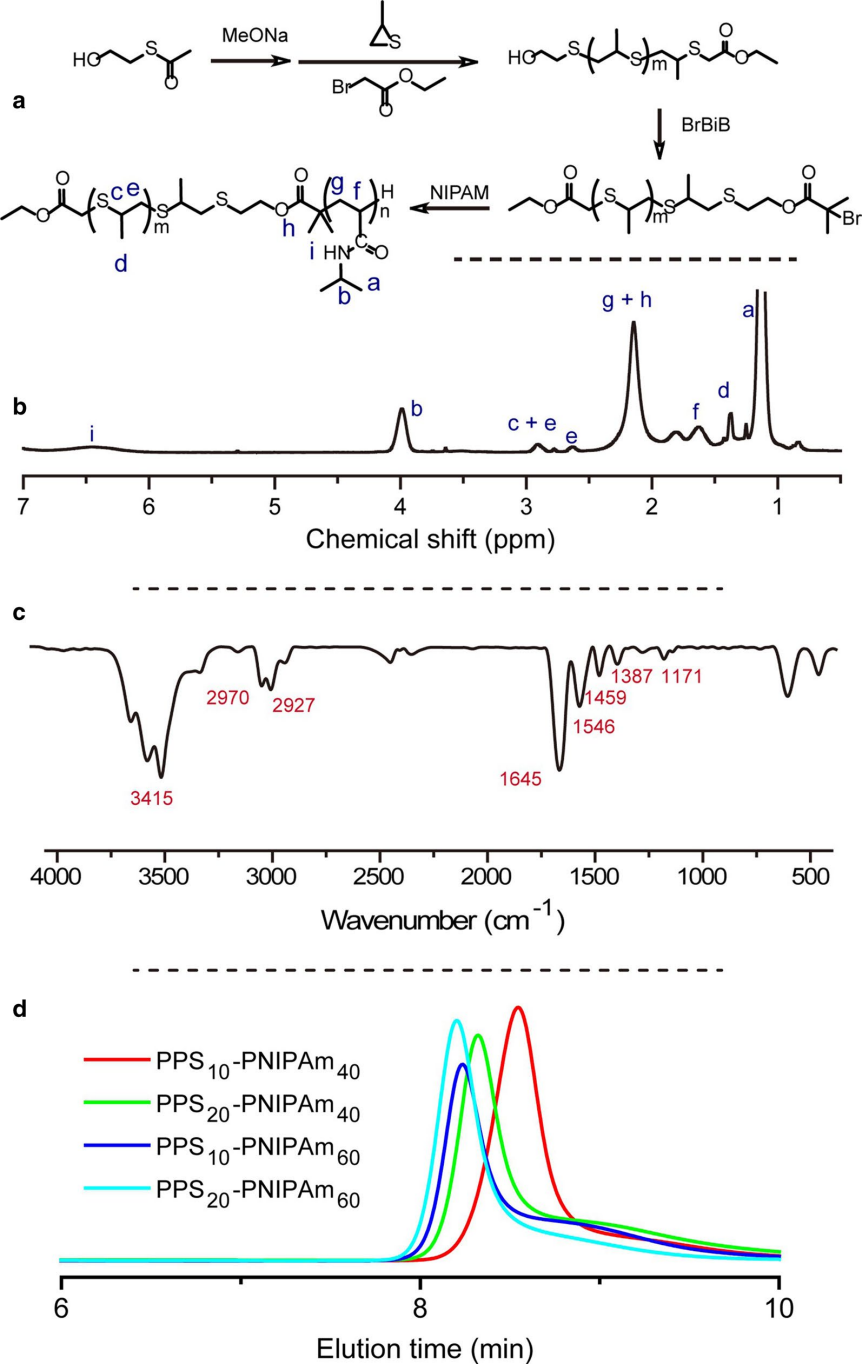
**a** Synthesis scheme of PPS-PNIPAm block copolymer and typical, **b** ^1^H-NMR, **c** FT-IR spectrum and **d** GPC traces of synthesised polymers

PPS-PNIPAm block copolymers were synthesised by living anionic ring-opening polymerization combined with an atom transform radical polymerization (ATRP) process as previously reported (Fig. 1a) [26]. PPS blocks with different polymerization degrees (PD) were synthesised followed by conversion of the terminal hydroxyl group into a bromide group. The resulted functional PPS block polymer was used as a macroinitiator to start an ATRP process of NIPAm, resulting in a small library of PPS-PNIPAm block copolymers. The synthesised polymers were characterized with ^1^H-NMR, FT-IR and GPC (Fig. 1; Additional file 1: Table S1). A typical ^1^H-NMR spectrum of PPS_10_-PNIPAm_40_ showed the chemical shifts corresponding to both PPS and P(NIPAm-*co*-DMAA) segments (c, d, e for PPS block, a, b, g and f for PNIPAm block, Fig. 1b). The molar ratio of PS and NIPAm monomers calculated by NMR was 9.6:42.2 (Additional file 1: Table S1), which agrees well with their theoretical molar ratio (10:40). FT-IR spectrum also showed the presence of both PPS and PNIPAm (Fig. 1c). Furthermore, the molecular weight distribution of the copolymers were determined by GPC measurement (Fig. 1d) and the values using poly(methyl methacrylate) as standard were listed in Additional file 1: Table S1. The narrow polydispersity indexes (Đ) of the polymers (1.09–1.18, Additional file 1: Table S1) further proved the success of the polymer synthesis.

### Preparation and characterizations of polymeric micelles

**Fig. 2:**
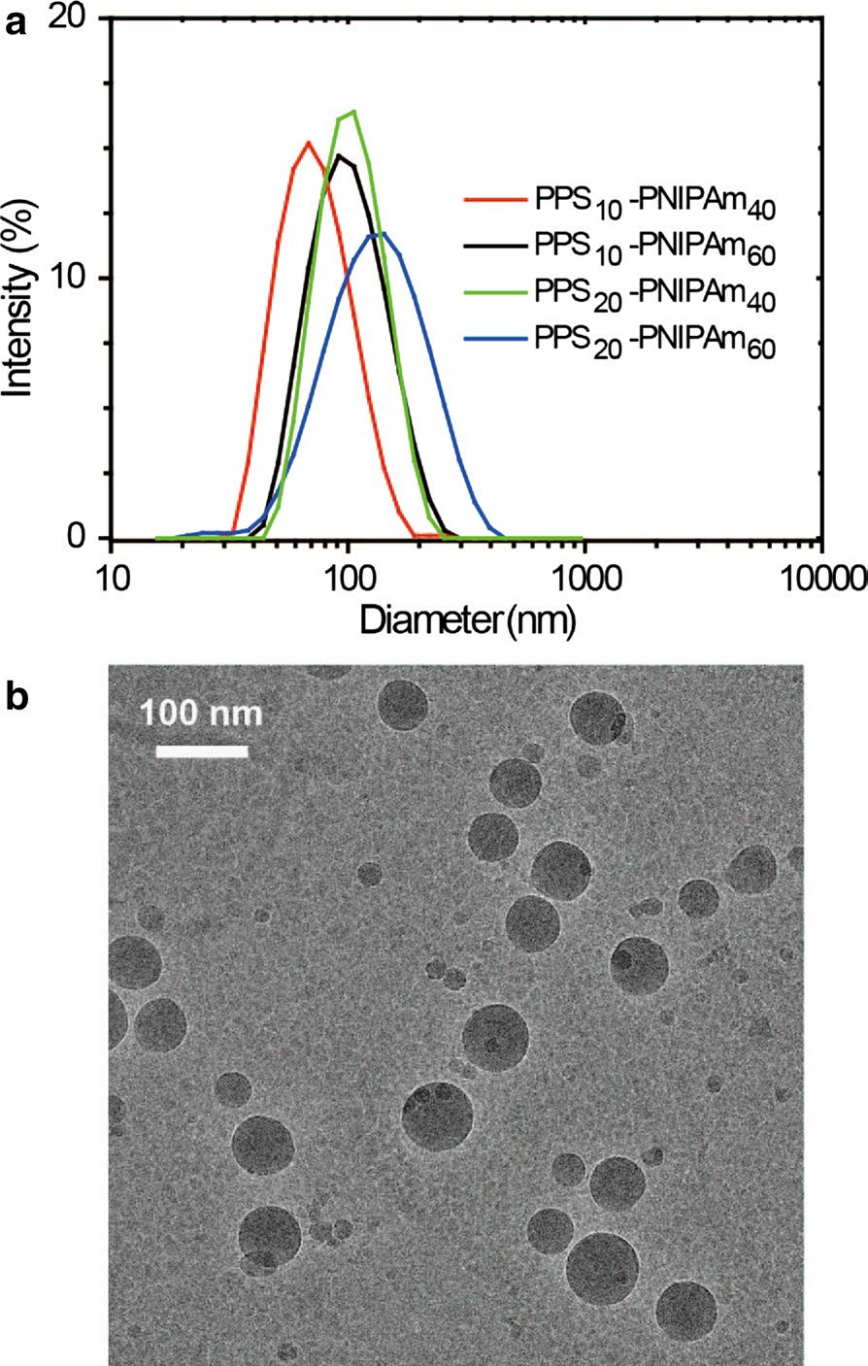
Characterization of PPS-PNIPAm micelles. **a** Size distribution of micelles formed with different block copolymers measured by DLS. **b** Cryo-TEM image of PPS_10_-PNIPAm_40_ micelles

Being amphiphilic of its nature, PPS-PNIPAm forms aggregates in aqueous solutions, presumably in nanoscales. Employed with a solvent–insolvent method, the polymers can easily form micellar aggregates. The CAC values scaled with the overall hydrophilicity of the polymers, ranging from 0.021 mg/mL for the most hydrophobic P3 to 0.0158 and 0.217 mg/mL for the most hydrophilic P2 and P4 (Additional file 1: Figure S1). Since the size and morphology affect the nanocarriers during the circulation and accumulation in the target sites, the micelles were further characterised by DLS and Cryo-TEM. Figure 2a shows PPS-PNIPAm formed micelles with sizes in the range of 70–110 nm, depending on the chain lengths of the polymers. For example, the average hydrodynamic size of PPS_10_-PNIPAm_40_ micelle was around 70 nm, compared to the larger particles (110 nm) formed by PPS_20_-PNIPAm_60_. Cryo-TEM clearly demonstrated the uniform spherical shapes of PPS_10_-PNIPAm_40_ micelles, whose observed diameters were consistent with the DLS results (Fig. 2b). Zeta-potential measurements showed the micelles bear a slightly negative surface charge of −6.98 mV (Additional file 1: Figure S3), which could allow its circulation in blood without overly captured by macrophageal and reticuloendothelial system [42, 43]. Overall, the nanoscale system prepared by PPS-PNIPAm polymer possesses suitable size and surface charge allowing its circulation in blood and accumulation in tumoral site through EPR effect.

**Fig. 3:**
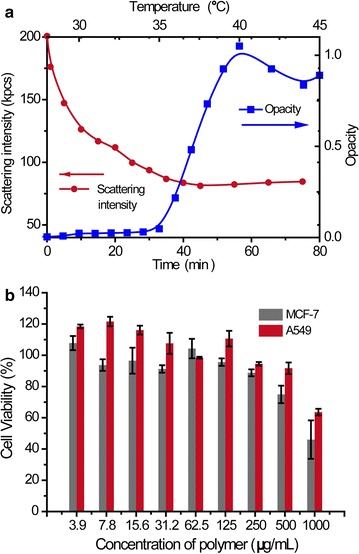
**a** Thermal responsiveness of PPS_10_-PNIPAm_40_ micelles characterized by increased opacity upon heating; Oxidation-responsiveness characterized with decreased scattering intensity upon addition of oxidants. **b** Biocompatibility of the polymer tested on MCF-7 and A549 cancer cell lines with concentrations up to 1000 μg/mL

The biocompatibility of the polymer was tested on A549 and MCF-7 cell lines. The results showed negligible toxicities of the polymer with concentrations up to 500 μg/mL (Fig. 3b).

### Dual responsiveness of polymeric micelles

The polymeric micelles demonstrated dual responsiveness. Upon heating, the turbidity of PPS-PNIPAm micelles dispersion increased drastically when being heated to around 35° (Fig. 3a), allowing the determination of the LCST to be 35.9°–37.1°, depending on the different structures of the polymers (Additional file 1: Figure S2). The oxidation-responsiveness was demonstrated by incubating the PPS_10_-PNIPAm_40_ micelles with 5% hydrogen peroxide and monitoring the effects by DLS. As shown in the Fig. 3a, the scattering intensity of the sample decreased drastically over 2 h, which could be due to the dissolution of predominantly oxidized colloids and the reduced scattering intensity of partially oxidised particles [8, 9].

### DOX loading and in vitro drug release

**Fig. 4:**
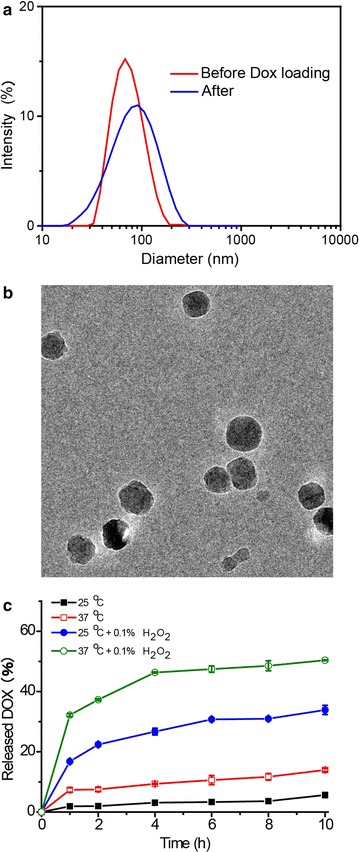
**a** Size distribution of PPS_10_-PNIPAm_40_ micelles before (*red*) and after (*blue*) drug loading. **b** Cryo-TEM image of DOX loaded PPS_10_-PNIPAm_40_ micelles. **c** In vitro release profile of DOX loaded micelles under different conditions: 25 °C (*black*), 37 °C (*red*), 25 °C + 0.1% H_2_O_2_ (*blue*) and 37 °C + 0.1% H_2_O_2_ (*green*)

Doxorubicin was used as a model drug molecule to be encapsulated in the PPS_10_-PNIPAm_40_ micelles with moderate encapsulation efficiency and drug loading (Additional file 1: Figure S4). DOX-loaded PPS-PNIPAm micelles showed a size distribution centred at 80 nm, slightly broader than the blank micelles (Fig. 4a). Figure 4b showed the solid spherical shapes of DOX loaded micelles observed by Cryo-TEM. The DOX release from the drug loaded micelles could be easily monitored because the fluorescence of encapsulated DOX is partially quenched, and the fluorescence can be restored after the DOX is released into the medium (Additional file 1: Figure S5). As shown in Fig. 4c, in the absence of oxidants, the release of DOX from the micelles was negligible up to 24 h at 25 °C or under heating at 37 °C. On the other hand, in the presence of 0.1% H_2_O_2_, DOX-loaded micelles demonstrated significant release of DOX, with a 22% release at 2 h and 31% release at 10 h. Furthermore, the drug release of the sample in the presence of two stimuli (37 °C and 0.1% H_2_O_2_) was even faster than under oxidation only, with a 38% release at 2 h and 51% release at 10 h. This could be explained as that the shrunk PNIPAm corona under 37 °C allowed an easier access of H_2_O_2_ to the PPS core and enhanced oxidation-responsiveness, resulted in such synergistic release profile of the PPS-PNIPAm micelles under two stimuli.

### Uptake of DOX-loaded micelles

**Fig. 5:**
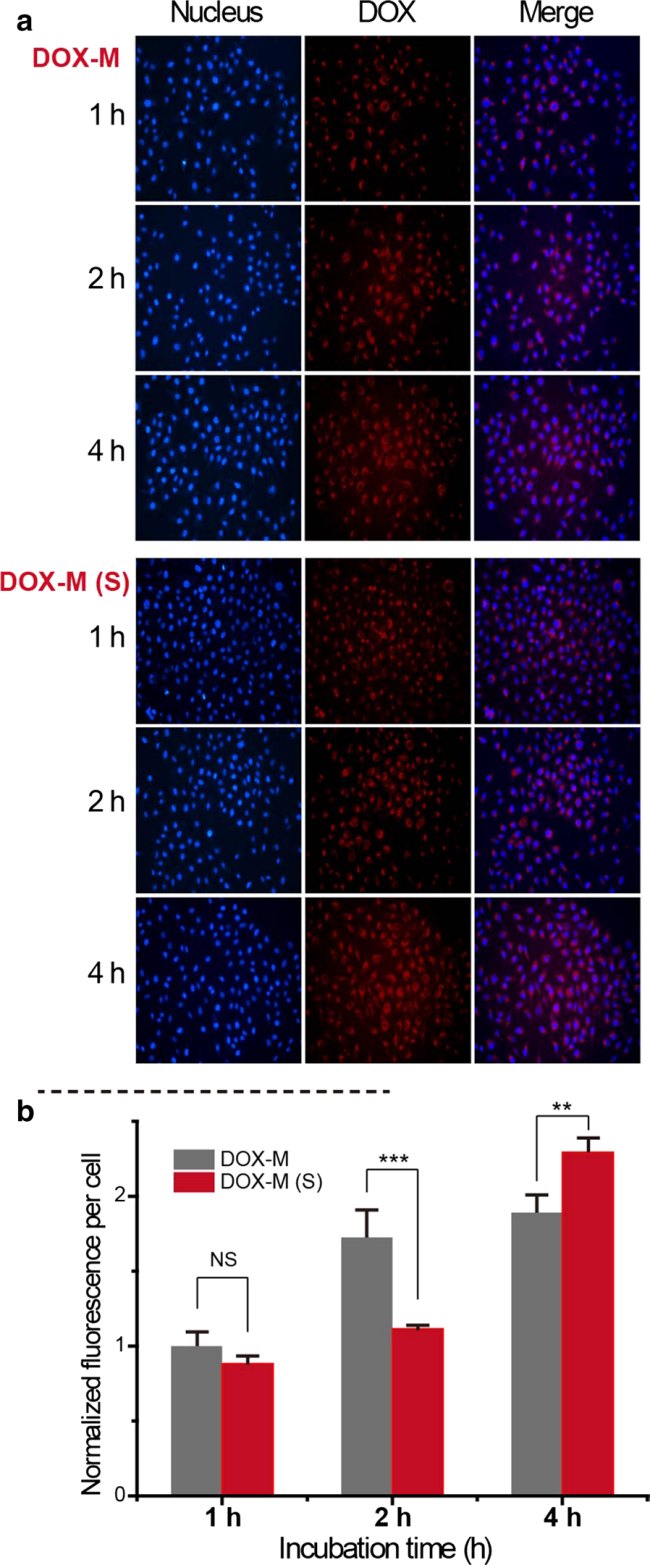
**a** Uptake of DOX loaded micelles (DOX-M) on MCF-7 cells. The photos were taken with a fluorescent microscopy. Cells pre-treated to produce more ROS were incubated with drug loaded micelles (DOX-M(S)). The nuclei were stained as *blue*, localization of DOX was *red* and their merged pictures were also showed. **b** Normalized fluorescence per cell analysed by Photoshop software (n = 3)

**Fig. 6:**
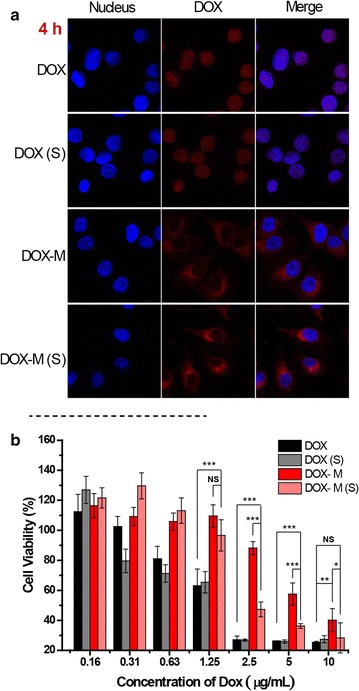
**a** CLSM images of the uptake of free DOX and DOX loaded micelles at 4 h. **b** The cytotoxicity of free DOX and DOX loaded micelles on MCF-7 cells

Cell internalisation experiments were performed to investigate the uptake of DOX-loaded micelles into MCF-7 cancer cells. Localisation of the drug molecules was observed via fluorescence microscopy and CLSM. As shown in Fig. 5a, the red fluorescence of DOX was weak after 1 h incubation and then gradually increased at 2 and 4 h, indicating a time-dependent uptake of the micelles into cells. At a higher magnification (Fig. 6a), it is apparent that at 4 h free DOX entered the cells and overlapped with stained nuclei, suggesting the binding of DOX to DNA in the nuclei (Fig. 6a, DOX). On the other hand, the DOX loaded micelles were taken up by cells and mainly remained in the cytoplasm (Fig. 6a, DOX-M). To further investigate the influence of ROS to the internalisation and intracellular release of the DOX, MCF-7 cells were treated with Rosup reagent (50 μg/mL) for 20 min to produce more ROS (Additional file 1: Figure S6) before incubation with samples. As shown in Fig. 5b, stimulated cells incubated with free DOX (DOX (s)) showed no substantial difference of internalisation to the non-stimulated cells, although the stimulated cells incubated with DOX-M (Fig. 6a, DOX-M (s)) showed higher intensity of DOX around the nuclei, which is possibly due to the relatively higher intracellular ROS concentration. However, the low intensity of nuclear DOX suggests that most of it might be not in a free form.

### Cytotoxicity study of DOX loaded PPS-PNIPAm micelles

The in vitro cytotoxicity of the DOX-loaded micelle was evaluated by determining the MCF-7 cell viability with a CCK-8 assay. Notably, the cytotoxicity of free DOX increased along with the concentrations with a half lethal dose (IC_50_) of 1.8 μg/mL at incubation time of 48 h (Fig. 6b). Free DOX showed higher toxicity than its micellar form possibly due to its fast diffusion into cell nuclei to effect the growth of the cells, while encapsulated DOX will have to be effective after it is released from the micelles upon the oxidation of PPS domain. It is therefore necessary to investigate the influence of ROS to the cell viability. A mild 20 min ROS stimulation was applied to the MCF-7 cancer cell, which itself does not influence the viability of the cells (Additional file 1: Figure S7). After 48 h of the incubation, the ROS stimulated cells demonstrated a significant difference of viability when treated with same concentrations of DOX-M, comparing to non-ROS stimulated cells. Particularly, the IC_50_ value was lowered from around 5 to about 2 μg/mL (Fig. 6b). This phenomenon was not observed on free DOX, indicating ROS generation only played a role when DOX was loaded in an oxidation-sensitive carrier.

## Conclusions

In conclusion, this study demonstrated a successful synthesis of PPS-PNIPAm block copolymers by combining living anionic ring-opening polymerisation and ATRP. The obtained polymer was utilized to construct a dual stimuli-responsive drug delivery platform for enhanced treatment. Such a polymeric delivery system was able to efficiently respond to temperature and ROS overproduction in cancer cells and correspondingly, release the encapsulated cargos inside cells with ROS generation in vitro. This strategy provides new insights into the design of thermally and oxidation responsive polymeric vesicles for DOX loading and its delivery to ROS overproduction related cancer cells.

## Additional file

**Additional file 1.** Supplementary material.

## Abbreviations

TBP: tris-*n*-butylphosphine
PS: propylene sulfide
DBU: 1,8-diazabicyclo [5.4.0] undec-7-ene
EBA: ethyl bromide acetate
BrBiB: 2-bromo-2-methylpropionylbromide
NIPAm: *N*-isopropyl acrylamide
DOX·HCl: doxorubicin hydrochloride
H_2_O_2_: hydrogen peroxide
ROS: reactive oxygen species
