# Supplementary material for: Polymeric micelles with dual thermal and reactive oxygen species (ROS)-responsiveness for inflammatory cancer cell delivery
Source: J Nanobiotechnology. 2017 May 16;15:39. doi: 10.1186/s12951-017-0275-4 (PMC5434630; doi:10.1186/s12951-017-0275-4)
Supplement: Supplementary file 1 — Additional file 1. Supplementary material. [file 12951_2017_275_MOESM1_ESM.docx]

**Supporting Information**

**Polymeric Micelles with Dual Thermal and Reactive Oxygen Species (ROS)-Responsiveness for Inflammatory Cancer cell Delivery**

Meiqiong Tang^a^, Ping Hu^a^*, Qiang Zheng^a^, Nicola Tirelli^b^, Xiaohong Yang^a^, Zhanlong Wang^a^, Yanfang Wang^c^, Qing Tang^a^, Yun He^a^*

^a^. School of Pharmaceutical Sciences, Chongqing University, 55 South Daxuecheng Road, Chongqing 401331, China

^b^. NorthWest Centre of Advanced Drug Delivery (NoWCADD), School of Pharmacy, and Centre for Tissue Injury and Repair, Institute of Inflammation and Repair, University of Manchester, Oxford Road, Manchester, M13 9PT, United Kingdom

^c^. First Affiliated Hospital of the Medical College, Shihezi University, Xinjiang 832008, PR China

**Corresponding authors:**

ping.hu@cqu.edu.cn, inzahu@hotmail.com, yun.he@cqu.edu.cn

**List of Contents:**

1. Summary of the results for polymers prepared through ATRP

2. Critical Aggregation Concentration (CAC) Determination

3. Lower Critical Solution Temperature (LCST) Determination

4. Zeta potential measurement

5. Drug Loading and Encapsulation Efficiency

6. Fluorescence spectra of micellar dispersion sample under oxidation

7. ROS determination on different cells

8. Cell viability after ROS stimulation

**1. Summary of the results for polymers prepared through ATRP**

*Table S1.* Summary of the results for polymers prepared through ATRP.

|  | **DP of PPS** | |  | **DP of PNIPAm** | |  | $\overline{M_{n}}$ | |  | **Đ ^e)^** | **LCST^f)^** | **CAC^g)^** |
| --- | --- | --- | --- | --- | --- | --- | --- | --- | --- | --- | --- | --- |
|  | **Theor.** | **Cal. ^a)^** |  | **Theor.** | **Cal. ^b)^** |  | **NMR^c)^** | **GPC^d)^** |  |  | **(℃)** | **(mg/mL)** |
| **P1** | 10 | 9.6 |  | 40 | 42.2 |  | 5600 | 5300 |  | 1.13 | 37.1 | 0.044 |
| **P2** | 10 | 10.9 |  | 60 | 58.3 |  | 7800 | 7400 |  | 1.15 | 37.6 | 0.158 |
| **P3** | 20 | 18.2 |  | 40 | 41.7 |  | 6400 | 6200 |  | 1.09 | 36.9 | 0.021 |
| **P4** | 20 | 22.3 |  | 60 | 61.1 |  | 8900 | 7900 |  | 1.18 | 36.7 | 0.217 |

^a^ Calculated from the ratio of the ^1^H-NMR signal of the CH_2_ groups in PPS repeating units (CH_2_ at 2.55-2.75 ppm ) and signals of the initiator (CH_2_ at 2.6 ppm ). ^b, c^ Calculated from the ratio of the ^1^H-NMR signal of the CH groups in PNIPAm repeating units (CH at 4.01 ppm ) and signals of the PPS repeating units (CH_2_ at 2.55-2.75 ppm ). ^d^ Determined via GPC in DMF at 30℃ with poly(styrene) standards. ^e^ Dispersity index calculated as $\overline{M_{\text{w}}}$/$\overline{M_{n}}$; ^f^ Determined at concentration of 1 mg/mL. ^g^ Obtained through the Nile Red method.

**2. Critical Aggregation Concentration (CAC) Determination**

**
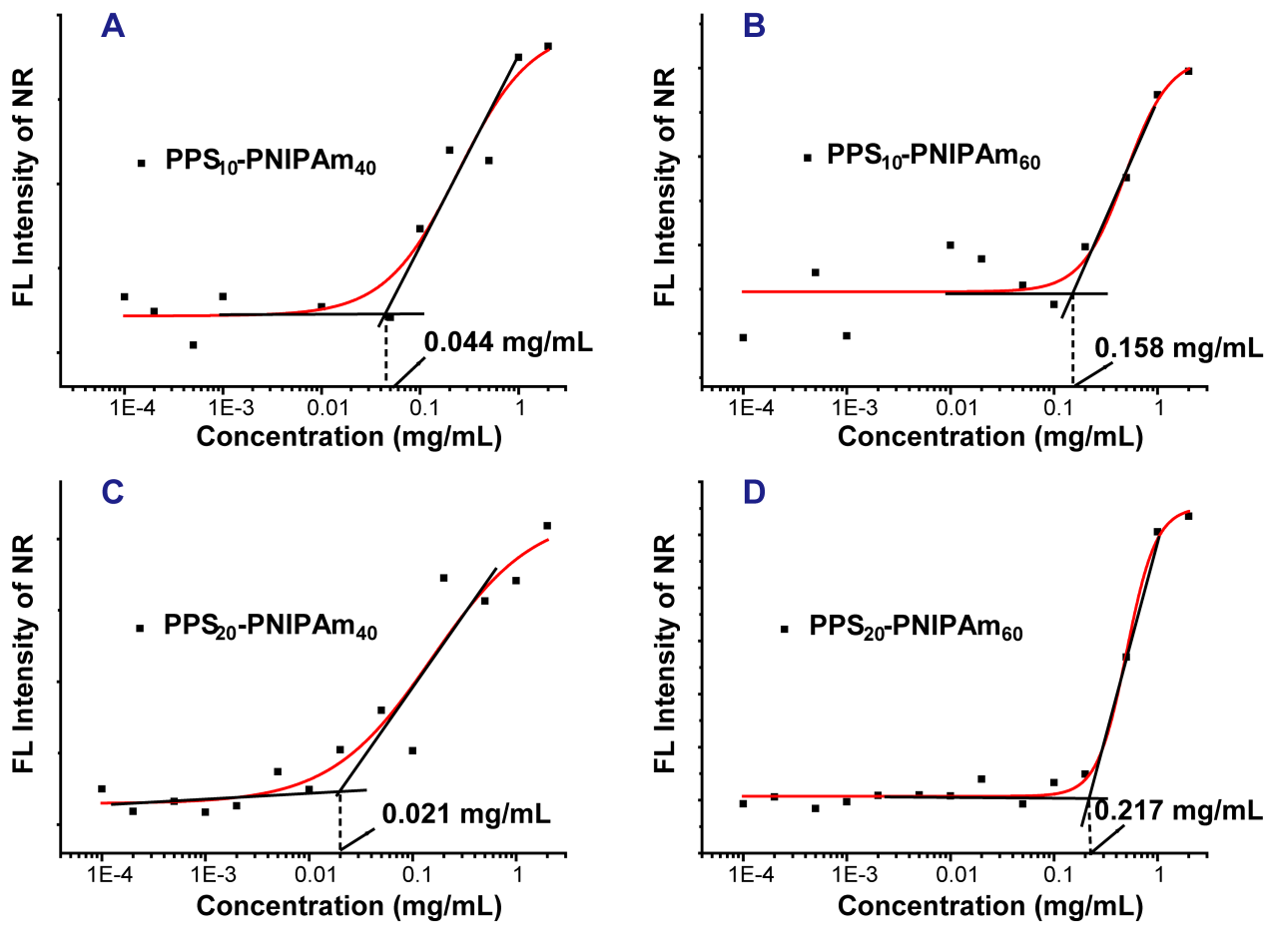
**

**Fig. S1.** CAC determination of PPS-PNIPAm micelles using Nile Red (NR) as fluorescent probe.

**3. Lower Critical Solution Temperature (LCST) Determination**

**
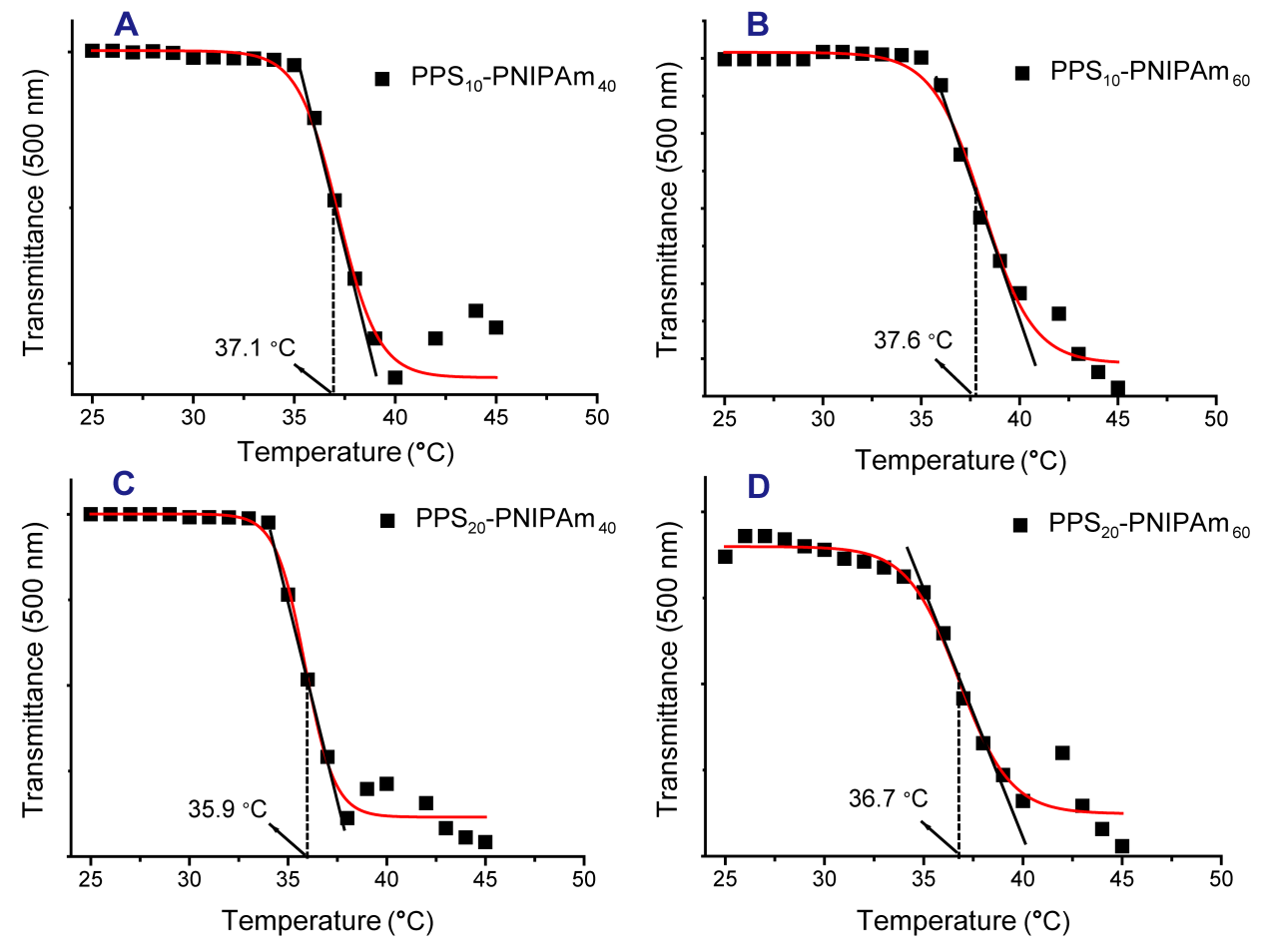
**

**Fig. S2.** LCST determination of PPS-PNIPAm in aqueous solution.

**4. Zeta potential measurement**





**Fig. S3.** Zeta potential of PPS_10_-PNIPAm_40_ micellar dispersions in pH 7.4 PBS buffer.

**5. Doxorubicin Loading and Encapsulation efficiency**


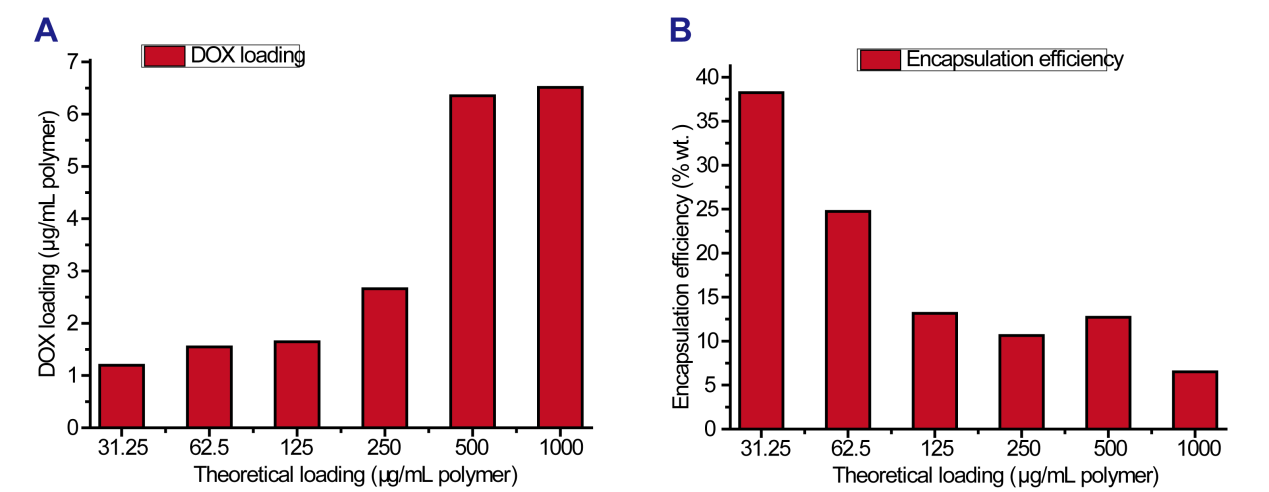


**Fig. S4.** Drug loading and encapsulation efficiency of PPS-PNIPAm micelles.

**6. Fluorescence spectra of micellar dispersion sample under oxidation**

**
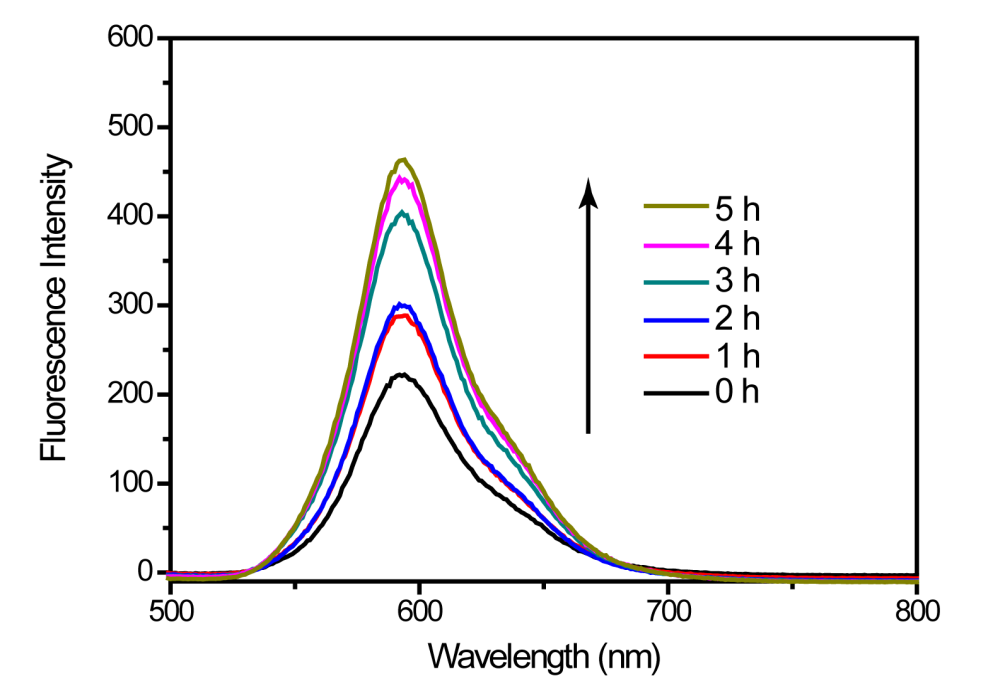
**

**Fig. S5.** Fluorescence spectra of DOX loaded micellar dispersion sample (1mg/mL of polymer) under oxidation (0.2% H_2_O_2_). The increase of fluorescence is due to the release of the DOX from the encapsulated form.

**7. ROS determination before and after stimulation**


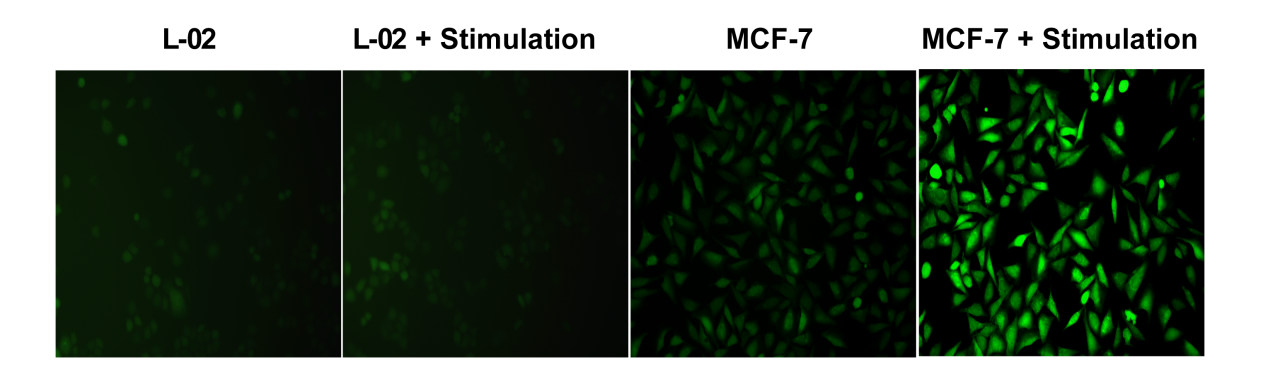


**Fig. S6.** ROS determination of L-02 and MCF-7 cells before and after stimulation.

DCFH-DA fluorescent probe was used to determine the ROS level in A549 and MCF-7 cell lines with different treatment. The A549 and MCF-7 cells were treated with Rosup (50 μg/mL) for 20 min. both of A549 and MCF-7 cells showed substantial ROS level and even higher ROS level after 20 min stimulation, which confirmed the suitability of the cell lines used as model. In addition, non-ROS rich human cells (L-02) showed no obvious ROS level comparing to cancer cells.

**8. Cell viability after ROS stimulation**


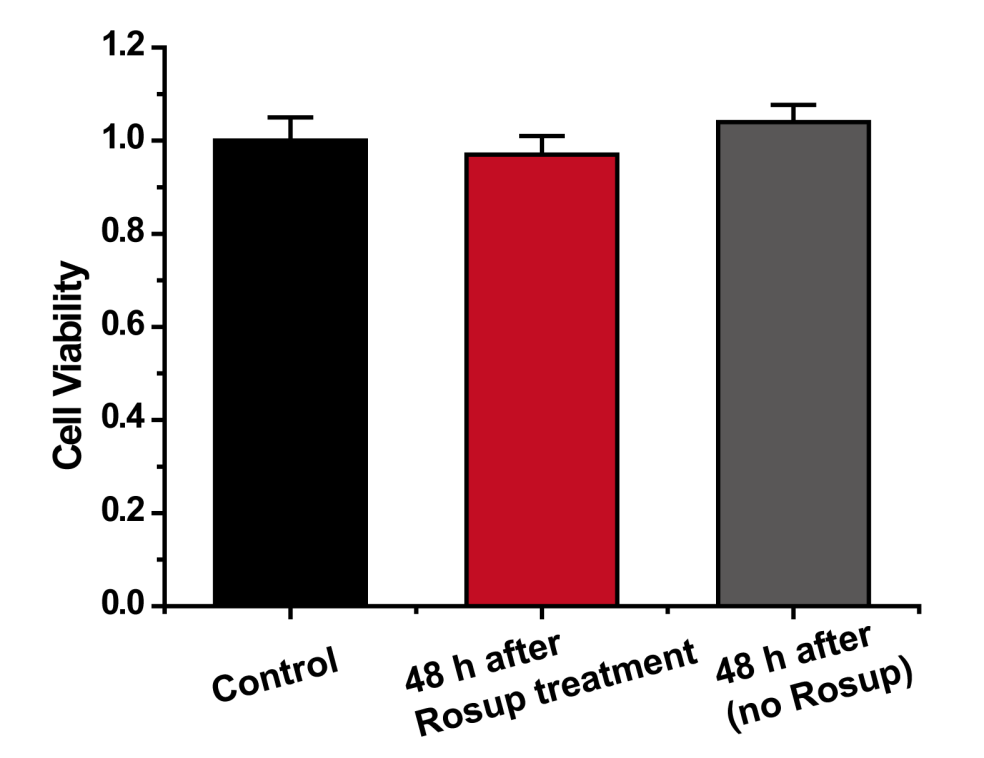


**Fig. S7.** Cell viability of MCF-7 cells with or without 20 minutes Rosup (50 μg/mL) treatment after 48 h incubation, using fresh cells without any treatment or incubation as control.
